# Development of a Solid Dispersion of Nystatin with Maltodextrin as a Carrier Agent: Improvements in Antifungal Efficacy against *Candida* spp. Biofilm Infections

**DOI:** 10.3390/ph14050397

**Published:** 2021-04-22

**Authors:** Carlos Benavent, Carlos Torrado-Salmerón, Santiago Torrado-Santiago

**Affiliations:** 1 Department of Pharmaceutics and Food Technology, Faculty of Pharmacy, Complutense University, Plaza Ramón y Cajal s/n, 28040 Madrid, Spain; carlosbenavent@ucm.es (C.B.); ctorrado@ucm.es (C.T.-S.); 2 Instituto Universitario de Farmacia Industrial, Complutense University, Plaza Ramón y Cajal s/n, 28040 Madrid, Spain

**Keywords:** solid dispersion, nystatin, amorphous form, maltodextrin, biofilms, *Candida albicans*

## Abstract

The aim of this study was to improve the treatment of *Candida albicans* biofilms through the use of nystatin solid dispersions developed using maltodextrins as a hyperosmotic carrier. Characterization studies by differential scanning calorimetry, X-ray diffraction, dissolution studies, and particle size analysis were performed to evaluate changes in nystatin crystallinity. Antifungal activity and anti-biofilm efficacy were assessed by microbiological techniques. The results for nystatin solid dispersions showed that the enhancement of antifungal activity may be related to the high proportions of maltodextrins. Anti-biofilm assays showed a significant reduction (more than 80%) on biofilm formation with SD-N:MD [1:6] compared to the nystatin reference suspension. The elaboration process and physicochemical properties of SD-N:MD [1:6] could be a promising strategy for treatment of Candida biofilms.

## 1. Introduction

In recent years, *Candida albicans* has become one of the most common agents associated with an adherent biofilm-forming microbial population [1]. These structures may appear on oral mucosa and on the surface of silicone or polyurethane materials used in different devices such as catheters and nasogastric or endotracheal tubes [2,3].

*Candida albicans* biofilms generally consist of a mixture of yeast, hyphae, and pseudohyphae surrounded by an extracellular polymeric matrix [4], which may act as a physical barrier [1,2]. Most antifungal agents are hydrophobic in nature and have a limited ability to penetrate this barrier [4]. Recent studies based on agar diffusion techniques have determined the relevance of antifungal penetration in biofilms formed in the oral cavity and clinical isolates of different *Candida* spp. [5]. Antifungal penetration has been shown to be effective for preventing biofilm formation and eliminating preformed biofilms in the oral cavity and on the surface of various medical devices [4,6]. The penetration of posaconazole and amphotericin B through biofilms was shown to be significantly reduced [4]. Modern antifungal strategies involving dendrimer therapies [7], magnetic nanoparticles [8], amphotericin solid dispersions [9], and nystatin (NYS) solid dispersions in chewing gum [10] have been demonstrated to prevent the formation of biofilms of different *Candida* spp. Medical device coatings have also shown enhanced antifungal activity when compared with antifungal suspensions [2,3,8].

Solid dispersions (SD) have been used to enhance the aqueous solubility of poorly water-soluble drugs [11]. This technique is based on the dispersion of poorly water-soluble drugs into a hydrophilic carrier, creating a dispersed state with improved solubility [12]. Solvent evaporation methods [13,14], spray drying methods [9], fusion [15], and fusion/extrusion [16] have been commonly employed for the production of solid dispersions. In many cases, the solubility increase for these systems was related to the crystallinity decrease by the inclusion of drug molecules within the carrier chains [14,17]. Solid dispersions with different antifungal active ingredients, such as NYS, voriconazole, or amphotericin B, have been designed to improve their solubility [9,10,18]. A study on the properties of the carrier is important for the elaboration of solid dispersions. Synthetic hydrophilic polymers, such as polyvinylpyrrolidone, polymetacrylates, polyvinyl alcohol and polyvinylacetate, improved drug wetting by dissolution and enhanced drug antimicrobial efficacy [19]. Semisynthetic derivatives, such as hydroxypropylcellulose or hydroxypropyl methylcellulose, were used with antifungal compounds such as itraconazole, clotrimazole, miconazole, and NYS [11,16]. Moreover, sodium croscarmellose [17], cyclodextrins [9,20], and maltodextrins (MDs) [21,22] increase the solid dispersion swelling and wettability of different antibiotics during dissolution essays.

In recent years, the combination of a high concentration of antibiotics with hyperosmotic agents has offered an alternative strategy for the treatment of biofilm communities [23,24]. Solid dispersions with different antifungal active ingredients, such as voriconazole or amphotericin B, have been designed to improve their solubility [9,18]. In addition, NYS solid dispersions have been developed with improved solubility in orodispersible tablets [25]. An increased efficacy against biofilm communities was observed with the osmotic effect of polyethylene glycol in antibiotic treatments, such as tobramycin, chloramphenicol [24], and NYS [10]. Voriconazole or clotrimazole solid dispersions with sugars such as mannitol, fructose, or dextrose showed an increased antifungal effect [14,15]. Furthermore, maltodextrin (MD), a hyperosmotic carrier, was studied with antifungals such as vancomycin or amphotericin B [21,26]. These hyperosmotic carriers increased the efficiency of antibiotic and antifungal agents in reducing biofilm coverage, thickness, and diffusion distance within the biofilm [21,24]. MDs also concealed the bitter flavor of some of these antifungal compounds due to their sweetness and fast dissolution, [27].

In this work, NYS solid dispersions were developed using MDs as hydrophilic carriers for the treatment and prevention of *Candida albicans* biofilms and studied by scanning electron microscopy (SEM), differential scanning calorimetry (DSC) and X-ray powder diffraction (XRPD) techniques. Dissolution tests determined the solid dispersions with faster dissolution profiles, and their activity was assessed in microbiological assays.

## 2. Results

Solid dispersions (SD) of nystatin (NYS) and maltodextrins (MD) at different proportions (SD-N:MD [1:1], SD-N:MD [1:4], SD-N:MD [1:6], and SD-N:MD [1:8]) were elaborated. This preparation resulted in a light-yellow colored powder similar to the corresponding physical mixture of NYS and MD (PM-N:MD). Characterization studies by SEM, FTIR, DSC, XRPD, and dissolution studies were carried out to evaluate the improvements of the different solid dispersions. In these studies, a formulation of NYS raw material and a physical mixture PM-N:MD [1:6] were used as references.

The different aqueous suspensions of the solid dispersions were reconstituted for the in vitro antifungal activity and prevention of biofilm formation assays. In these studies, the hyperosmotic effect of the solid dispersion SD-N: MD [1:6] was evaluated against a micellar system with sodium deoxycholate (DC) as a control. The non-osmotic character of the control micellar system MS-N:DC [1:0.8] allowed us to evaluate the hyperosmotic effect of the MD carrier.

### 2.1. Scanning Electron Microscopy (SEM)

**Figure 1:**
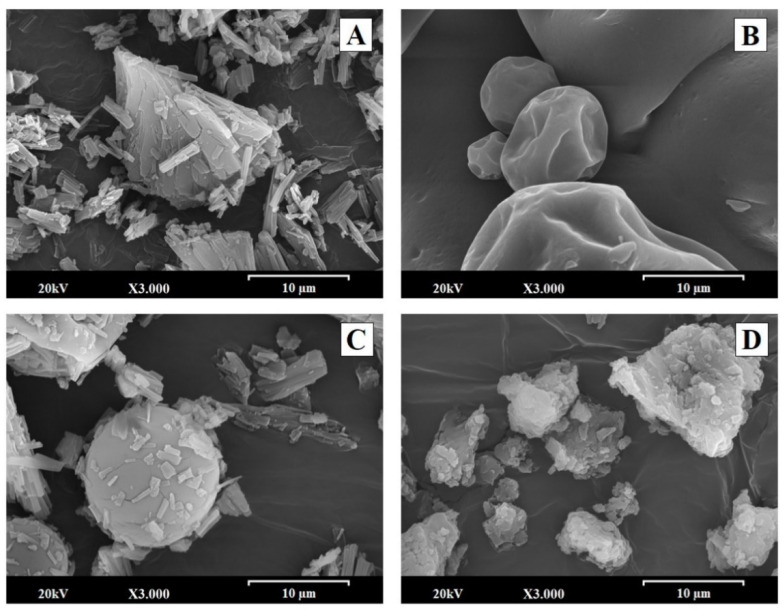
SEM micrographs of (**A**) surface-modified nystatin raw material (NYS); (**B**) maltodextrin (MD); (**C**) PM-N:MD [1:6] physical mixture and (**D**) SD-N:MD [1:6] solid dispersion. Photographs were taken at a magnification of 3000×.

The particle morphology, size, and shape were identified by SEM. Figure 1 shows microphotographs of the NYS raw material (Figure 1A), the MD raw material (Figure 1B), the PM-N:MD physical mixture [1:6] (Figure 1C), and the SD-N:MD [1:6] solid dispersion (Figure 1D) taken at a magnification of 3000×.

The NYS raw material (Figure 1A) showed the presence of small polyhedral crystals (3–6 µm), which tend to form aggregates. MD particles (Figure 1B) appeared as smooth, spherical, globular particles with a larger size (10–50 µm). The PM-N:MD [1:6] physical mixture had a high proportion of smooth spherical MD particles with small NYS crystals (3–6 µm) on its surface (Figure 1C).

The particle morphology changed substantially after the process of preparing the solid dispersions. SD-N:MD [1:1], with a low proportion of carrier, presented NYS crystalline aggregates of 10–20 µm with small MD particles on its surface (data not shown). However, SD-N:MD [1:6] (Figure 1D) showed the formation of a matrix structure of MD (5–20 µm) with no NYS crystallinity present.

### 2.2. FTIR Spectroscopy Analysis

**Figure 2:**
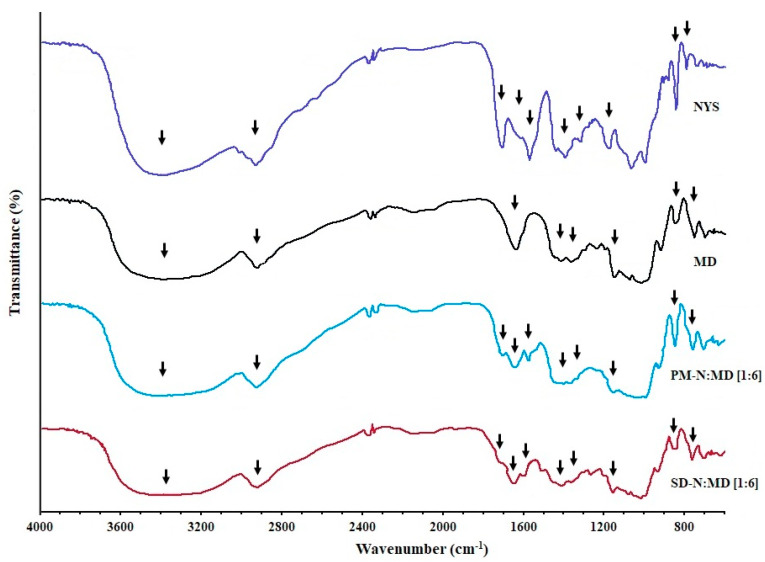
FTIR spectrum of nystatin raw material (NYS), maltodextrin (MD), PM-N:MD [1:6] physical mixture, and SD-N:MD [1:6] solid dispersion.

The FTIR spectra of the NYS raw material (NYS), pure maltodextrin (MD), the physical mixture of NYS with maltodextrin PM-N:MD [1:6] and the SD-N:MD [1:6] solid dispersion are presented in Figure 2.

The spectra of the NYS powder (Figure 2) showed a broad peak at 3367 cm^−1^, which could be assigned to O-H stretching vibration. The peak located at 2937 cm^−1^ was identified as the C-H_2_ stretch vibration. The peaks at 1709 and 1620 cm^−1^ were characteristic of C=O stretching vibrations in the carboxylic group and C=C asymmetric stretching, respectively. The peak at 1575 cm^−1^ was related to the C=C vibrations of heptane. The peaks at 1439, 1382, and 1175 cm^−1^ could be ascribed to C-O and C-O-H stretching vibrations. In addition, peaks at 1069, 848 and 795 cm^−1^ were observed, which can be attributed to =C-H, -CH2 and –C–H stretch vibrations [10,28].

MD powder showed similar peaks at 3367 and 2927 cm^−1^, characteristic of O-H and C-H_2_ stretching vibrations, respectively (Figure 2). The peak at 1644 cm^−1^ could be assigned to C=O stretching vibration. The intense bands at 1438, 1369, and 1157 cm^−1^ were characteristic of C=O stretching vibrations and C-O-H bending, respectively. The peak at 1017 cm^−1^ was due to the angular deformation of =C-H and =CH_2_ bonds. The peaks at 850 and 708 cm^−1^ were attributed to -CH_2_ and C-H stretching characteristics of the structural state of the pyranose ring frequencies in the pure MD structure [29,30].

The physical mixture PM-N:MD [1:6] showed two broad peaks at about 3403 and 2933 cm^−1^ which were characteristic of O-H stretching vibration. In these spectra (Figure 2), a decreasing intensity of the NYS peak at 1709 cm^−1^ was attributed to the dilution effect. The peaks at 1651 and 1578 cm^−1^ were characteristic of the C=O stretching vibration of MD. The peaks at 1404 and 1370 cm^−1^ could be ascribed to C-O and C-O-H stretching vibrations. The decreases in the intensity of both peaks at 848 and 763 cm^−1^ were attributed to the dilution effect and structural state of the pyranose ring frequencies in the pure MD structure.

SD-N:MD [1:6] exhibited two broad peaks at 3382 and 2929 cm^−1^, which were characteristic of O-H and C-H_2_ stretching vibrations, respectively, and were attributed to the high proportion of MD (Figure 2). The peaks at 1644 and 1623 cm^−1^ were related to the C=O stretching vibration and the C=C asymmetric stretching of NYS. The peaks at 1404 and 1369 cm^−1^ could be ascribed to C-O and C-O-H stretching vibrations, and the peaks at 1014, 849, and 762 cm^−1^ were associated to the –C-O-C stretching vibration and H–C–H stretch vibration bands [29,30].

### 2.3. Differential Scanning Calorimetry (DSC)

**Figure 3:**
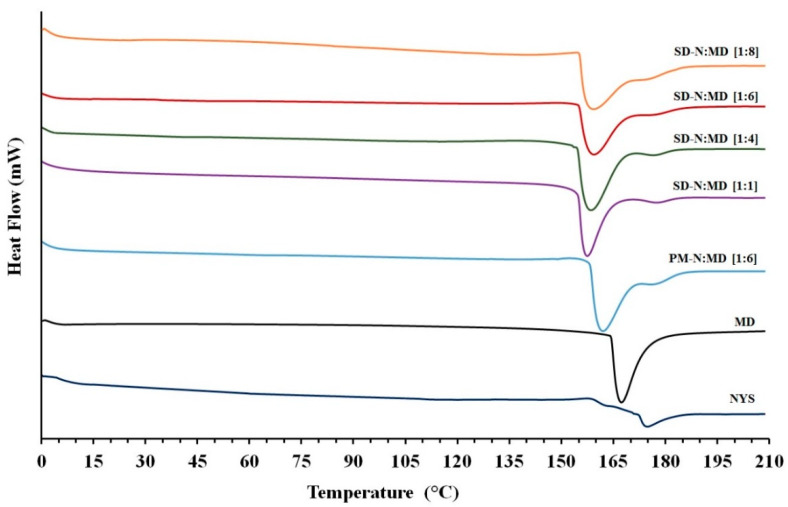
DSC thermograms of nystatin raw material (NYS), maltodextrin (MD), PM-N:MD [1:6] physical mixture, and SD-N:MD [1:1], SD-N:MD [1:4], SD-N:MD [1:6], and SD-N:MD [1:8] solid dispersions.

DSC studies (Figure 3) showed an endothermic peak for NYS at 174.28 °C with an enthalpy value of −111.85 J/g, associated with low crystallinity. The MD carrier exhibited a broad endothermic peak at 167.30 °C (−1272.59 J/g), characteristic of semicrystalline structures. PM-N:MD [1:6] presented characteristic endothermic peaks at 163.43 and 177.87 °C for MD and NYS, respectively. The high enthalpy of fusion of MD produced a shift in the endothermic peak of NYS, showing a higher temperature. The changes in the NYS peak were caused by NYS/MD interactions during the heating process.

Solid dispersions showed a shift at lower melting temperatures for the endothermic peak of MD (between 155.71 and 159.85 °C) with a significant decrease in their enthalpy values (Figure 3). SD-N:MD [1:1] and SD-N:MD [1:4] revealed a substantial decrease in the crystallinity of NYS, whereas SD-N:MD [1:6] and SD-N:MD [1:8] showed an amorphous structure for NYS.

### 2.4. X-ray Powder Diffraction (XRPD)

**Figure 4:**
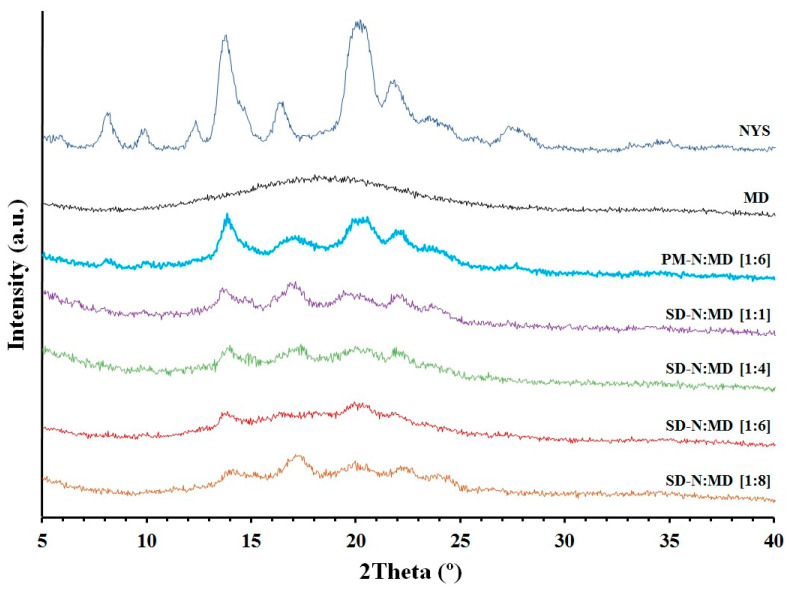
XRPD diffraction patterns of nystatin raw material (NYS), maltodextrin (MD), PM-N:MD [1:6] physical mixture, and SD-N:MD [1:1], SD-N:MD [1:4], SD-N:MD [1:6], and SD-N:MD [1:8] solid dispersions.

The XRPD patterns can be seen in Figure 4. The NYS raw material showed a diffractogram of a crystalline substance with diffraction peaks at 7.9°, 13.91°, 16.5° and 20.05° 2θ [16]. MD exhibited a characteristic semicrystalline halo between 11–26° 2θ (Figure 4) [31]. PM-N:MD [1:6] showed a decrease in the intensity of the NYS diffraction peaks due to the dilution effect of MD [17]. However, significant changes in the intensity of the peaks were observed in the solid dispersions. SD-N:MD [1:1] and SD-N:MD [1:4] showed a decrease in NYS crystallinity related to the diffraction peaks at 13.91° and 20.05° 2θ; similar results were observed in the DSC studies. SD-N:MD [1:6] and SD-N:MD [1:8] revealed an amorphous structure for NYS, and it was not possible to quantify the crystallinity for the NYS peaks.

### 2.5. Dissolution Study

**Figure 5:**
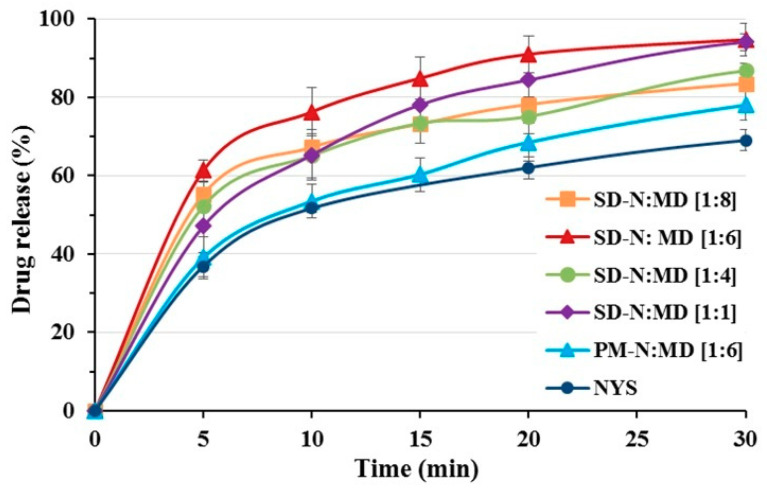
Dissolution profiles of nystatin raw material (NYS), PM-N:MD [1:6] physical mixture, and SD-N:MD [1:1], SD-N:MD [1:4], SD-N:MD [1:6], and SD-N:MD [1:8] solid dispersions.

The NYS raw material (Figure 5) presented a fast dissolution profile, which may affect its antifungal activity. The dissolution percentage was 36.87 ± 2.54% at 5 min, and attained levels of >50% at 10 min. The PM-N:MD [1:6] physical mixture (Figure 5) exhibited an improvement in the dissolution profile initially (5–20 min), with a slight increase at 5 min (39.05 ± 4.29%) compared to the NYS raw material.

NYS solid dispersions followed different dissolution patterns that were correlated with the proportion of MD. SD-N:MD [1:1] and SD-N:MD [1:4] (Figure 5) presented low dissolution profiles with percentages of 47.11 ± 5.84% and 52.22 ± 4.88%, respectively, at 5 min. No significant differences were observed with the physical mixtures initially. These percentages may be correlated with the NYS crystallinity reduction observed in the DSC and XRPD assays and the aggregation process observed during the dissolution studies. However, SD-N:MD [1:6] and SD-N:MD [1:8] (Figure 5) showed a significant increase (*p* < 0.05) at 5 min (61.35 ± 2.72 and 55.26 ± 3.21%, respectively) compared to PM-N:MD [1:6]. The absence of aggregation processes and the fast dissolution profiles led us to select SD-N:MD [1:6] as the correct formulation for microbiological tests.

### 2.6. Particle Size

The NYS formulation reached a large particle size of 650.50 ± 10.26 nm (99.6%), which was related to high percentages of aggregation as observed in SEM studies (see Figure 1). SD-N:MD [1:6] had a smaller particle size and a two-peak distribution at 53.14 ± 4.82 nm (49%) and 337.90 ± 26.06 nm (51%). A similar decrease in the particle size was showed by MS-N:DC [1:0.8], with values of 346 ± 18.41 nm (94.4%). The low particle sizes of SD-N:MD [1:6] and MS-N:DC [1:0.8] were related to a reduced aggregation process, which could allow NYS to remain in contact with the *Candida albicans* biofilms [32].

### 2.7. In Vitro Antifungal Activity Assay

**Figure 6:**
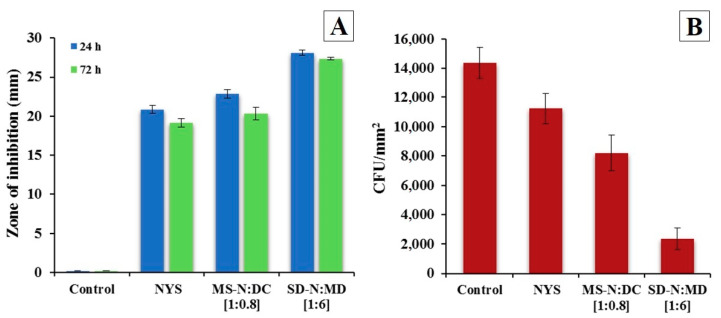
Effects of different formulations on *Candida albicans*. (**A**) Halo zone for fungal inhibition (mm) after 24 and 72 h. (**B**) *Candida albicans* adherence on plastic discs. Adherence is expressed as the number of colony-forming units per mm^2^ after incubation for 48 h. Formulations: control = nystatin raw material (NYS), nystatin micellar system MS-N:DC [1:0.8], and solid dispersion SD-N:MD [1:6].

As shown in Figure 6A, all NYS formulations showed good in vitro antifungal activity against *Candida albicans* (inhibition halo > 15 mm) [26]. After 24 h, the NYS aqueous solution showed a substantial inhibition of growth (20.84 ± 0.53 mm). The non-treated and MD discs had no inhibition area. However, the inhibition area of the NYS micellar systems MS-N:DC [1:0.8] presented a larger inhibition diameter (22.83 ± 0.55 mm), and was higher in solid dispersion SD-N:MD [1:6] (28.11 ± 0.37 mm), preventing the formation of aggregates [33]. All differences are statistically significant (*p* < 0.05). Solid dispersion maintained higher inhibition levels at 72 h.

Previous assays have demonstrated antifungal activity for NYS (median MIC: 128 μg/mL) [34]. The drug concentration applied on antibiotic discs is 15 mg/mL; as the discs have a capacity of 300 µL of fungal suspensions, the final amount of drug on the discs is 4.5 mg, so 10- or 100-fold dilutions of the solid dispersions have a NYS concentration above the median MIC. We therefore considered 15 mg/mL to be an adequate concentration to obtain therapeutic effects.

### 2.8. Biofilm Formation on Clinical Devices

As shown in our previous research [33], five rinses are required to eliminate unattached fungal colonies. Each rinse was isolated and harvested; fungal growth was revealed when sessile colonies were present. The fifth rinse showed no fungal colonies on the plate. The biofilm formation on the pieces was quantified, revealing a mean of 14,375.22 ± 1060.58 CFU/mm^2^.

### 2.9. In Vitro Assay of the Prevention of Biofilm Formation

Figure 6B shows the inhibition of *Candida albicans* adhesion on clinical devices. Biofilms were formed over non-treated pieces (control), with a mean of 14,375.22 ± 1060.58 CFU/mm^2^ counted. The NYS aqueous solution (NYS) revealed a reduction in the adherence of *Candida albicans* of about 20% compared with the control (*p* < 0.001). The micellar system MS-N:DC [1:0.8] allowed us to obtain a NYS solution that prevents aggregation (positive control), with inhibition values of over 42% of biofilm formation. SD-N:MD [1:6] was able to prevent *Candida albicans* adherence and biofilm formation more effectively, with an inhibition percentage of over 83% compared with the control, and was statistically significant (*p* = 0.0002).

## 3. Discussion

NYS solution is considered the reference treatment for oral candidiasis [26]. However, it has an erratic bioavailability in oral mucosa due to its poor aqueous solubility [32].

MDs are widely used to incorporate bitter-flavored drugs in the solid state as they mask unpleasant flavors [27,35]. Hyperosmotic carriers may also have a synergic effect with antibiotic agents against biofilms [23,24]. Although high proportions of MD in physical mixtures delay the initial release, this issue has been resolved by developing solid dispersions of NYS with saccharide compounds, which enhances its hydrosolubility in oral buccoadhesive tablets [25]. Improved hydrophilic properties and a reduction in particle size have been demonstrated in several studies on antifungal solid dispersions [14,15]. Our characterization assays confirm that the NYS solid dispersion shows fewer particle aggregates and a significant reduction in crystallinity.

Solid dispersions with high proportions of carrier may also reduce the aggregation of crystals in hydrophobic drugs, enhancing water uptake and antifungal activity [14]. The absence of NYS crystals on the surface of MD in the SD-N:MD [1:6] solid dispersion has been correlated with a molecular dispersion, with NYS particles included inside the MD matrix (Figure 1D). Previous assays have demonstrated the ability of hydrophilic excipients to form a matrix structure capable of including hydrophobic compounds [22,36]. The tendency of NYS to aggregate due to its hydrophobic properties has been observed in SEM images. Previous works have indicated that aggregation processes may reduce the solubility of hydrophobic antifungal agents such as voriconazol [14].

In the FTIR studio, the spectrum of SD-N:MD [1:6] was quite similar to that of PM-N:MD [1:6] and no appreciable peak disappearance or changes in wave number were observed. This result indicated that there were no functional group interactions, and only the proportionally weaker intensity of the absorption band as the proportion of the MD carrier increased. In addition, the broad peaks between 3200–3500 cm^−1^, could be assigned to the O-H stretching of inter- or intra-molecular interactions, indicating that hydrogen bonding has an important role in the molecular interactions between NYS and MD [29,30].

The characteristic endothermic peaks of NYS confirmed the crystallinity observed in previous studies [37]. The broad endothermic peak for MD was attributed to crystalline regions produced by the absorption of water on the particle surface [38]. The shift to higher temperatures for NYS in the PM-N:MD [1:6] physical mixture indicated a phase-change behavior and was related to a displacement during the heating process [32]. The decrease in NYS values observed in the DSC and XRPD studies for PM-N:MD [1:6] was due to the dilution effect with different semi-crystalline excipients [17]. During the preparation of solid dispersions with low proportions of hydrophilic carriers SD-N:MD [1:1], MD was unable to incorporate all the drug molecules, which explains the NYS crystallinity observed in DSC and XRPD studies. The significant decrease in NYS crystallinity for SD-N:MD [1:4] was associated with the high rate of uptake of NYS hydrophobic molecules within the hydrophilic structure. Similar decreases in crystallinity have been observed with excipients such as mannitol [15] and MD [23]. Amorphous forms of NYS were observed for SD-N:MD [1:6] and SD-N:MD [1:8], and were explained by the uptake of NYS inside the MD complex [7]. The decrease in the semicrystalline structure of the MD in SD-N:MD [1:6] and SD-N:MD [1:8] was confirmed with DSC and XRPD studies, and attributed to the links between the hydrogen groups in the water molecules and the hydroxyl groups in the carbohydrate during the drying process of the solid dispersions [38,39]. These hydrophilic interactions prevent the agglomeration of NYS particles [17,39].

The NYS and PM-N:MD [1:1] dissolution profiles are correlated with NYS particle size and the crystallinity observed in the DSC and XRPD assays [10].

The SD-N:MD [1:1] solid dispersions revealed low dissolution percentages at 5 min, owing to the recrystallization and aggregation of NYS. Solid dispersions with low proportions of hydrophilic vehicles favored crystalline agglomerates of NYS and delayed its initial release [5,17]. The high dissolution percentage at 5 min (>60%) observed for SD-N:MD [1:6] compared to the NYS raw material was due to its amorphous shape and the lower aggregation, as shown in the particle size and SEM studies [39].

Recent assays have revealed an enhancement in the antifungal activity of griseofulvin when formulated as a solid dispersion [13]. Our initial hypothesis, that the hyperosmotic effect of MD would increase the antifungal effect of NYS, has been confirmed. A significant increase in antifungal activity of SD-N:MD [1:6] was observed in comparison with a NYS control solution. Biofilms are complex structures with protection mechanisms that include slowing the diffusion of antifungal agents [4]; the hyperosmotic ability of our formulations to prevent *Candida albicans* adherence and biofilm formation is a highly promising strategy for fungal biofilm treatments [24].

Our research consists of a novel method to simulate biofilm formation on clinical devices [33]. All NYS formulations were able to prevent biofilm formation in vitro. However, significant differences were seen when these formulations were tested on clinical material: the NYS aqueous solution reduced adhesion and biofilm formation by slightly over 20%. This result also indicates poor drug penetration into the biofilm matrix due to the high crystallinity and hydrophobicity of the antifungal agent [4,5,20]. The increment in the inhibition effect with the SD-N:MD [1:6] solid dispersion (fungal adhesion was inhibited by more than 80%) is well correlated with the results obtained from SEM, DSC and XRPD analyses. The improvement of the water solubility of NYS and the reduction in particle size allows for the greater diffusion of the drug in the biofilm and inhibits its formation. MD has also been shown to increase the osmotic effect of the formulation, which destabilizes the biofilm, allowing the penetration of antibiotics and enhancing its potency against pathogens [24]. Similar effects have been demonstrated against bacterial biofilms with combinations of vancomycin and MD [21]. Further research with different osmotic carriers or changing the proportions of MD is recommended.

## 4. Materials and Methods

### 4.1. Materials

Nystatin (NYS) was provided by Fragon Ibérica (Barcelona, Spain). *N*,*N*-dimethylacetamide (DMAc), sodium chloride, and glucose were purchased from PanReac AppliChem ITW Reagents (Barcelona, Spain). Maltrodextrin-Glucidex® (MD) was obtained from Roquette (Lestrem, France). Agar Sabouraud, Mueller–Hinton agar (MHA), yeast nitrogen base medium (YNBM), and yeast extract peptone dextrose (YPD) were provided by Difco Laboratories Inc. (Franklin Lakes, NJ, USA). Silicone nasogastric tubes were provided by Mallinckrodt Medical (Athlone, Ireland). The water used in these studies was obtained from a Milli-Q water purification system (Billerica, MA, USA). All reagents and chemicals used were of analytical grade.

### 4.2. Methods

#### 4.2.1. Preparation of the Formulations

NYS raw material (NYS-RM) and the PM-N:MD 1:6) physical mixture were used as references for characterization (SEM, XPRD and DSC) and dissolution studies.

To prepare the PM-N:MD [1:6] physical mixture, 375 mg of NYS and 2250 mg of MD hydrophilic vehicle ([1:6] *w*/*w*) were weighed; this formulation was prepared by manually mixing the two components in a ceramic bowl using a polymeric spatula. The physical mixture was sieved between 0.297 and 0.850 mm. The mixture was yellow in color because NYS was added in the solid state.

NYS solid dispersions SD-N:MD [1:1], SD-N:MD [1:4], SD-N:MD [1:6] and SD-N:MD [1:8] (*w*/*w*) were prepared by adding 375 mg of NYS to 1000 µL of DMAc and dissolving by vortexing (FisherbrandTM; Milan, Italy) at 2500 rpm for 2 min. Different proportions of MD were added to the NYS solution and mixed in a ceramic bowl. The formulations were dried at 40 °C for 72 h and sieved between 0.297 and 0.850 mm.

The micellar system MS-N:DC [1:0.8] was employed as a NYS non-hypertonic control solution. This formulation was prepared by adding 375 mg of NYS to 1000 µL of aqueous solution containing 300 mg of sodium deoxycholate (DC). The solution was agitated for 2000 rpm for 2 min. The formed micellar solution was dried at 40 °C for 24 h and sieved to achieve a particle size fraction of 0.297–0.850 mm. Different aqueous preparations were elaborated with NYS raw material, the physical mixture, and solid dispersions containing 15 mg/mL of NYS.

#### 4.2.2. Scanning Electron Microscopy (SEM)

Samples were mounted and sputtered under vacuum with a thin gold–palladium layer using a sputter coater metallizator (Q150RS; Quorum Technologies, Laughton, UK). After the coating process, a Jeol JSM-6400 scanning electron microscope (Tokyo, Japan) was used to analyze the samples, and the micrographs were taken at an accelerating voltage of 20 kV. All images of the surface morphology of the samples were captured at a magnification of 3000×.

#### 4.2.3. FTIR Study

Fourier transform infrared (FTIR) spectroscopy of NYS raw material (NYS), pure maltodextrin (MD), the physical mixture of NYS with MD (PM-N:MD [1:6]) and the SD-N:MD [1:6] solid dispersion were performed with FTIR-8400 S (Shimadzu®; Kioto, Japan). Samples were mixed with KBr powder (2:100) and compressed into 10 mm discs. The scanning range was 400–4000 cm^−1^ with a spectral resolution of 4 cm^−1^.

#### 4.2.4. Differential Scanning Calorimetry (DSC)

Samples were mounted on a TC 15 thermal analyzer (Mettler Toledo®; Schwerzenbach, Switzerland). The temperature was calibrated using the Indium Reference Standard. Samples were accurately weighed into aluminum pans and heated from 20 °C to 250 °C at a rate of 10 °C/min under constant purging of dry nitrogen at 20 mL/min. A sealed empty pan was used as a reference for each sample analyzed.

#### 4.2.5. Powder X-ray Diffraction (XPRD)

X-ray diffraction patterns were recorded using a Philips® X’Pert-MPD X-ray diffractometer (Malvern Panalytical®; Almelo, Netherlands) with a 30 kV voltage and 30 mA current. Each sample was irradiated with monochromatized CuKα radiation (λ = 1.542 Å) and data on the diffraction patterns in the range between 5° and 50° 2θ degrees was collected and scanned at a step size of 0.04° and a time of 1 s per step. These studies were performed in the CAI XRD technological research center (Centro de Asistencia a la Investigación, UCM, Madrid, Spain).

#### 4.2.6. Dissolution Study

The dissolution study was performed with ERWEKA DT 80 (ERWEKA GmbH; Langen, Germany) dissolution equipment with a speed of 100 rpm and 600 mL of PBS medium adjusted to a pH of 4.5 (USP42-MF37, 2019). The United States Pharmacopeia (USP) paddle method (apparatus 2) was used as a guideline. A temperature of 37 ± 0.5 °C was maintained in all the tests in the study.

Various NYS solid dispersions SD-N:MD [1:1]; SD-N:MD [1:6], and SD-N:MD [1:8] were assayed with 10 mg of NYS in each formulation. NYS raw material and PM-N:MD [1:6] were used as controls. Each quantity was placed on the dissolution vessels, and samples were collected at the times specified in the USP. After filtering through a 0.45 µm filter (Acrodisc^®^, Port Washington, NY, USA), the remaining NYS was determined at 306 nm with a UV-VIS JASCO V-730 spectrophotometer (Jasco^®^ International Co., Ltd.; Tokyo, Japan), with the following calibration curve: y = 0.0647 × (µg/mL) −0.0002 (r^2^ = 0.9985) across a range of 1–15 µg/mL. Each test was performed in triplicate.

#### 4.2.7. Particle Size Analysis

The particle size of the microcapsule powder was determined using a laser particle size analyzer (Microtrac S3500, Microtrac Inc., North Largo, FL, USA). The reconstituted solutions NYS and SD-N:MD [1:6] were diluted, and the particle size and percentage of particles for the different solid dispersions was expressed in nanometers (nm).

#### 4.2.8. Antifungal Assays

In vitro antifungal activity was assayed with a technique based on the agar diffusion assay, tested on *Candida albicans* (CECT 1394). Fungal cells were cultured on petri plates with Sabouraud dextrose agar for 72 h at 36 °C (±2 °C). Fungal colonies (1 × 10^6^ cell per mL) were suspended on sterile phosphate buffered saline (PBS) (1X PBS) and applied in sterile petri dishes. Each plate contained a blank disc impregnated with an MD aqueous solution as a negative control and a micellar system of NYS and sodium deoxycholate MS-N:DC [1:0.8] as a NYS solution (positive control). After 24 and 72 h of incubation at 37 °C, the inhibition halo was determined on each plate. All experiments were prepared in triplicate.

#### 4.2.9. Biofilm Formation on Clinical Devices

The adhesion of antifungal drugs on the surface of clinical devices is a promising strategy for the treatment and prevention of biofilm formation [4,40]. The fungal biofilm was formed with the experimental technique based on Maki’s method [33], consisting of contaminating 1 cm^2^ of sterile silicone Mallinkroft nasogastric tubes with 12 mL of 0.5 Macfarland standard *Candida albicans* suspension containing 1 × 10^6^ CFU/mL for 48 h at 37 °C. Control and blank plates were also harvested. The pieces were gently rinsed five times with NaCl 9‰ after harvesting to remove most of the non-adhered planktonic organisms. The pieces were placed in 10 mL tubes with 3 mL of NaCl 9‰, and then sonicated and vortexed twice for 1 min at 1500 rpm. Then, 50 µL was harvested on petri plates with Sabouraud dextrose agar for 48 h at 37 °C. After this period, fungal colonies were counted, and the biofilm-forming colonies were quantified.

#### 4.2.10. In Vitro Assay: Prevention of Biofilm Formation

The active formulations and pieces of non-contaminated devices were kept in contact for 3 min before performing the biofilm formation assay. A reduction in colony-forming units (CFU) after harvesting indicated the preventive activity of biofilm formation. An aqueous solution of NYS and SD-NYS:MD [1:6] at 15 mg/mL were tested. The control plates contained pieces of the device that had no contact with the active formulations to ensure the formation of a biofilm. Furthermore, a micellar system with a [1:0.8] (*w*/*w*) ratio of NYS and sodium deoxycholate was assayed as a NYS solution MS-N:DC [1:0.8] (positive control) [33]. Negative controls were obtained from blank dishes with non-contaminated solvents.

## 5. Conclusions

In this study, surface-engineered solid dispersions of NYS and MD were successfully developed and assessed. NYS crystallinity was observed in solid dispersions with low proportions of the MD carrier: SD-N:MD [1:1] and SD-NYS:MD [1:4]. However, amorphous NYS forms appeared in SD-N:MD [1:6]. Additionally, high proportions of MD reduced the aggregation of NYS particles in solid dispersions compared with NYS raw material. Improvements in MIC for SD-N:MD [1:6] were linked to a high proportion of MD carrier. The greater anti-biofilm effect seen for SD-N:MD [1:6] compared to NYS alone was correlated with the reduction in particle size and the increased hyperosmotic effect of MD in these systems.

## Data Availability

Not applicable.
